# Application of Artificial Intelligence for Bridge Deterioration Model

**DOI:** 10.1155/2015/743643

**Published:** 2015-10-22

**Authors:** Zhang Chen, Yangyang Wu, Li Li, Lijun Sun

**Affiliations:** Key Laboratory of Road and Traffic Engineering of the Ministry of Education, Tongji University, Shanghai 201804, China

## Abstract

The deterministic bridge deterioration model updating problem is well established in bridge management, while the traditional methods and approaches for this problem require manual intervention. An artificial-intelligence-based approach was presented to self-updated parameters of the bridge deterioration model in this paper. When new information and data are collected, a posterior distribution was constructed to describe the integrated result of historical information and the new gained information according to Bayesian theorem, which was used to update model parameters. This AI-based approach is applied to the case of updating parameters of bridge deterioration model, which is the data collected from bridges of 12 districts in Shanghai from 2004 to 2013, and the results showed that it is an accurate, effective, and satisfactory approach to deal with the problem of the parameter updating without manual intervention.

## 1. Introduction

In bridge management, many problems, especially in bridge deterioration model, were influenced by uncertainties which not only could be solved in need of mathematics and mechanics calculations but also depend on the knowledge and experience of experts [1, 2]. The most interesting condition of the bridge is the performance, which is evaluated by studying the functional or structural behavior of the bridge. Bridge deterioration model is one of the key elements of bridge performance, which can be used to analyze bridge life-cycle costs and estimate the type and timing of bridge maintenance and rehabilitation needs. In these models, the dependent variable of observed or measured structural or functional deterioration is related to a set of independent variables. There are two broad categories of models available in the literature: deterministic models and probabilistic models. Deterministic models estimate the average value of the dependent variable (such as the remaining life of a bridge or its level of distress). Most deterministic models used in bridge deterioration model are based on regression analysis [3–5]. Probabilistic models estimate a range (or distribution) of values for a dependent variable. Most probabilistic models used in bridge deterioration model are based on Markovian theory [6–8].

Bridge deterioration model, especially deterministic models, need to update the parameter constantly when new data is collected. The conventional regressive analysis would consume a large number of computation time and computing resources. What is more, it relies heavily on the manual intervention in bridge management. However, artificial intelligence has its own superiority. It can solve this problem by means of imitate human intelligence.

Artificial intelligence (AI) is generally considered to be a subfield of computer science that is concerned to attempt simulation, extension, and expansion of human intelligence [9, 10]. It was developed based on the interaction of several kinds of disciplines, such as computer science, cybernetics, information theory, psychology, linguistics, and neurophysiology [11]. The goal of this field is to explore how to imitate and execute some of the intelligence functions of human brain, so that people can develop technology products and establish relevant theories [12, 13]. The nominal birth of AI is considered to have occurred at a conference held at Dartmouth College in the summer of 1956. After that, AI has been a far-reaching cross-frontier subject. Its tools and techniques are in the mainstream of computer science and at the core of so many systems, such as expert system, intelligence database system, and knowledge base system. We have made significant headway in solving fundamental problems in representing knowledge, reasoning, machine learning, and more, and a lot of hopes and dreams about it have been generated [14–18]. Artificial intelligence has also been widely applied to update model parameters. Corchado and Aiken [19] presents the application of a novel hybrid artificial-intelligence model to a real time forecasting problem, which provided an effective strategy for forecasting in an environment in which the raw data is derived from three distinct sources. Amayri and Bouguila [20] proposed a principled statistical framework that simultaneously determines relevant features, number of clusters while being able to incrementally update model's parameters. Mishra [21] used* H*_∞_-learning method for updating the parameter of the radial basis function neural network to improve the transient stability performance of a multimachine power system. Khodaparast et al. [22] adapted a similar method and proposed two perturbation based methods to determine the first and the second statistical moments of updating parameters. They investigated the influence of correlation between the updated and the modal parameters on the results of a 3-degree-of-freedom simple system and truss structure. Moaveni et al. [23] presented a study in order to quantify and analyze uncertainties in damage detection problem using finite element model updating strategy. They perform a gradient based optimization method to minimize the objective function which includes the residuals of experimental and numerical modal parameters for different combinations of uncertainty source. Singhal and Kiremidjian [24] used Bayesian analysis method for updating earthquake ground motion by damage relationships and for estimating confidence bounds when building damage information from historical earthquakes becomes increasingly available. Enright and Frangopol [25] applied Bayesian method updating to condition prediction of deteriorating concrete bridge, which combined the information both from inspection result and from engineering judgment, and this developed approach was illustrated for a reinforced bridge. Strauss et al. [26] proposed a Bayesian approach for updating prediction functions, which incorporated past data and information. Ching and Leu [27] proposed a Bayesian algorithm to quickly update the reliability of the infrastructure system according to the timely condition data, and this method was demonstrated with a real-world case of hydraulic spillway gate system. Wang et al. [28] developed a Bayesian approach for updating a model for predicting excavation settlement using centrifuge test information, and the results of case study showed that the accuracy can be improved.

An artificial-intelligence-based approach (AI-based approach) for updating deterministic model parameters of bridge deterioration model is researched in this paper. From the above literatures, it can conclude that the characteristics of Bayesian theorem are well suited for updating models, while the process of model updating cannot work without manual intervention. For this problem, an AI-based approach for updating deterministic model parameters is proposed by using Bayesian theorem in this paper, which imitate some of intelligence function of human brain and realize the self-updating parameters of bridge deterioration model.

In the first section, we overview the bridge deterioration model and artificial intelligence, especially Bayesian theorem. In Section 2, the framework of AI-based approach for updating parameters is founded, and then the mechanism of AI-based approach for updating parameters is proposed, which is the core of AI-based approach for updating parameters and can be applied to realize the parameter self-updating of bridge deterioration model. In Section 3, a case study about bridge deterioration model is illustrated for the process of the AI-based approach for updating parameters. We then summarize the proposed AI-approach in Section 4.

## 2. AI-Based Approach

### 2.1. The Framework of AI-Based Approach for Updating Parameters

AI-based approach for updating parameters allows bridge deterioration model to self-update parameters when new information or data is collected using Bayesian theorem. Mathematically, the Bayesian theorem can be expressed as follows:

(1) $$\begin{array}{c} p\left(\;\alpha_{1}\;|\;\alpha_{2}\;\right)=\frac{f\left(\alpha_{2}\;|\;\alpha_{1}\right)\pi \left(\alpha_{1}\right)}{\int_{\Theta }f\left(\alpha_{2}\;|\;\alpha_{1}\right)\pi \left(\alpha_{1}\right)d\alpha_{1}}, \end{array}$$

where *π*(*α*_1_) is the prior distribution of model parameters, which describes the historical information and data before any new information or data of *α*_2_ is obtained. In other words, *π*(*α*_1_) represents the initial parameters, and it is acceptable to a skeptical scientific audience. *f*(*α*_2_∣*α*_1_) is the likelihood function, which describes the sampling results about any new information or data of *α*_2_. *p*(*α*_1_∣*α*_2_) is the posterior distribution, which describes the integrated result of historical information *π*(*α*_1_) and the new information *f*(*α*_2_∣*α*_1_). In fact, the resulting posterior distribution is always a compromise between the prior credibility of the parameter values and the likelihood of the random variables for the data. This compromise is increasingly controlled by the data as the sample size increases in what is sometimes referred to as asymptotic theory [29–31].

**Figure 1:**
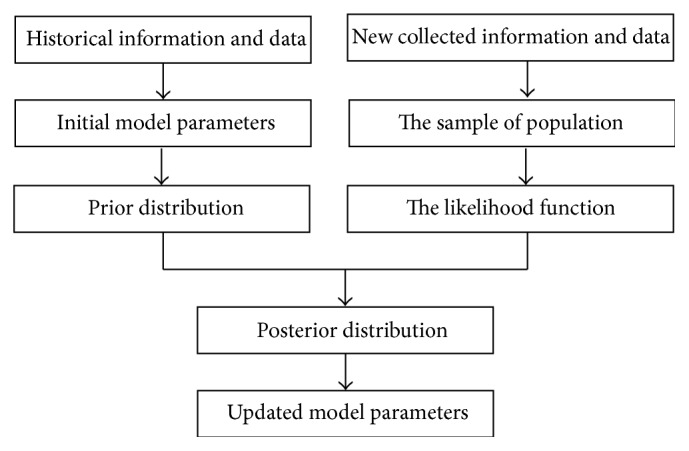
The framework of AI-based approach for updating parameters.

In this AI-based approach for updating parameters, the updating process can be divided into three stages. Firstly, a prior distribution should be established. A large amount of historical information and data are used to quantify as the prior distribution, namely, the initial model parameters. Secondly, a posterior distribution should be established when time series data is obtained in real-time, which includes the information of both the prior distribution and new collected data. Finally, model parameters can be updated by the posterior distribution. And Figure 1 resumes this overall updating process.

### 2.2. The Mechanism of AI-Based Approach for Updating Parameters

From the framework of the AI-based approach for updating parameters, the historical and new collected information and data are integrated and depicted by the posterior distribution, so it is the emphasis and difficulty of how to construct a posterior distribution *p*(*α*_1_∣*α*_2_) based on the prior distribution *π*(*α*_1_) and new collected data *X* as (1). In this section, the parameters of bridge deterioration model were regarded as unknown variables; namely, the mean and the variance were random unknown variables, and the derivation process from a prior distribution to a posterior distribution will be discussed in detail. When the sample size is large enough, it is reasonable that a normal distribution is used to describe the parameters of bridge deterioration model according the center limit theorem [32]. In this AI-based approach for updating parameters, the initial parameters of bridge deterioration model was set based on the historical data, the new obtained data was regarded as a sample of the population, and then we can deduce a posterior distribution about the parameters of bridge deterioration model according to the prior distribution and the sample.

Assuming that population follows a normal distribution *N*(*α*, *δ* ^2^), which consists of historical data and new obtained data, the value of population parameter (*α*) can be gained according to the least square method. The historical data also follows a normal distribution *N*(*α*_1_, *δ*_1_^2^), parameter (*α*_1_) can be gained by regressing those historical data, and it is used to quantify as the prior distribution of population parameter (*α*). The new collected data is regarded as a sample of the population and also follows a normal distribution *N*(*α*_2_, *δ*_2_^2^), and parameter (*α*_2_) can be gained by regressing the new collected data. Before any new information and data are collected, we consider the following:

(2) δ21=δ12,α1=α1.

According to the Bayesian theorem, a connection is constructed between the historical information and new collected information, so parameters *α* and *δ* ^2^ are calculated, and the formulations are given as [33]:

(3) δ22n1δ12+n2δ22n1+n2+n1n2n1+n22α1−α22≈n1δ12+n2δ22n1+n2,

(4) α2α1/δ12+α2/δ21/δ12+1/δ2=α1δ22+α2δ12δ22+δ12,

where *n*_1_ is the size of the historical data, parameters *α*_1_ and *δ*_1_^2^ are gained by regressing the historical data, *n*_2_ is the size of the new collected data, and parameters *α*_2_ and *δ*_2_^2^ are gained by regressing the new collected data.

In next stage, the new data will be collected continually. In this case, this new collected data is regarded as a sample of the population, and any other information and data become historical information and data, which is descripted by the prior distribution. It is obvious that this prior distribution equals the posterior distribution at the previous stage. In other words, for (3), the size of the historical information and data should be assigned by the sum of (*n*_1_) and (*n*_2_). For (4), the parameter *α*_1_ should be assigned by *α*. It is as follows:

(5) δ23=n1δ12+n2δ22+n3δ32n1+n2+n3,α23=α2δ23+α3δ22δ22+δ23.

From the above description and inference, the recursion formulas are constructed for updating model parameters when new information and data are gained continually. They are given as follows:

(6) δ2i=n1δ12+n2δ12+⋯+niδi2n1+n2+⋯+ni=∑j=1injδj2∑j=1inj,i=1,2,3,…,

(7) αi=α1,i=1,αi−1δ2i+αiδ2i−1δ2i−1+δ2i,i=2,3,4,…,

where *i* is the times of updating model parameters and other notations are the same as above.

According to (6) and (7), the new collected information and data are entered into parameters on the basic of the information and data gathered in the previous stage, so the model parameters can be self-updating consistently.

## 3. Approach Verification through Case Study

The AI-based approach for updating parameters is tested and verified through bridge deterioration model with the data collected from bridges of 12 districts in Shanghai from 2004 to 2013. The bridge deterioration model that was proposed by Chen of Tongji University in 2005 is currently adopted by bridge management agencies in Shanghai. The model form is as follows [34]:

(8) $$\begin{array}{c} \text{PPI}={\text{PPI}}_{0}\left\{\;1-\exp\left[\;-{\left(\;\frac{\alpha }{y}\;\right)}^{\beta }\;\right]\;\right\}, \end{array}$$

where PPI is the index of bridge performance, such as BCI; PPI_0_ is the index of initial bridge performance; *y* is the age of bridge; and *α* and *β* are the model parameter. It is necessary to update *α* and *β* consistently because of the changes in the external environment and the structure of the bridge itself.

The regressive results of new collected data are listed in Table 1.

The current approach (hereafter referred to as “conventional approach”) updates the parameters by regressing all data once new data is collected. For example, in 2004, the value of parameters *α* and *β* can be gained by regressing the data collected from 2004, the regressive results are *α* = 22.0, *β* = 1.09, and the size of processed data is 124. In 2005, the value of parameters *α* and *β* is gained by regressing the data collected from 2004 to 2005, the regressive results are *α* = 21.7, *β* = 1.07, and the size of processed data is 264, which is the sum of the size of the data collected from 2004 to 2005. On the analogy of this, in 2013, the value of parameters *α* and *β* is gained by regressing the data collected from 2004 to 2013, the regressive results are *α* = 22.8, *β* = 0.99, and the size of processed data is 1725, which is the sum of the size of the data collected from 2004 to 2013.

The AI-based approach developed in this paper (hereafter referred to as “new approach”) updates the parameters according the formulations (7) and (8). In this Approach, the model parameters can be directly calculated on the basic of the parameter of original data and the parameter of new collected data, instead of regressing the population of original data and new collected data. For example, it can be found that the value of parameter is as follows from Table 1: *α*_2004_ = 22.0, *β*_2004_ = 1.09, *δ*_2004_^2^ = 0.390, *n*_2004_ = 124, *α*_2005_^1^ = 21.58, *β*_2005_^1^ = 1.14, *δ*_2005_^12^ = 0.316, and *n*_2005_^1^ = 140. Afterward, according formulations (7) and (8), it is easy and quickly to get the value of the parameter in 2005. The calculated process is as follows:

(9) δ20052n2004δ20042+n20051δ200512n2004+n20051=124×0.390+140×0.316124+140=0.351,α2005α2004δ20052+α20051δ20042δ20052+δ20042=22.0×0.351+21.58×0.3900.351+0.390=21.8,β2005β2004δ20052+β20051δ20042δ20052+δ20042=1.09×0.351+1.14×0.3900.351+0.390=1.12.

**Figure 2:**
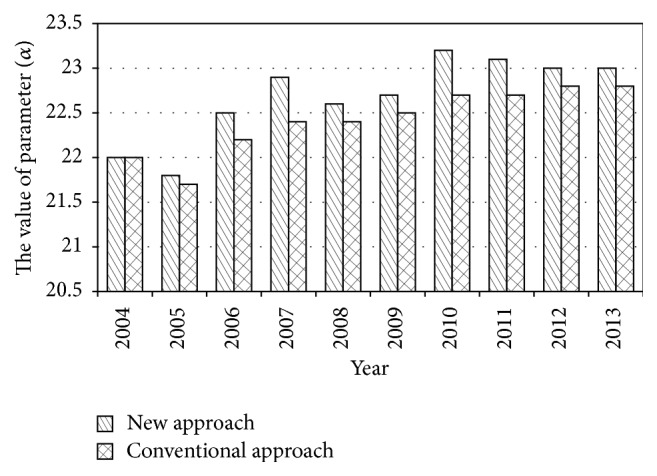
The value of parameter (*α*).

**Figure 3 fig3:**
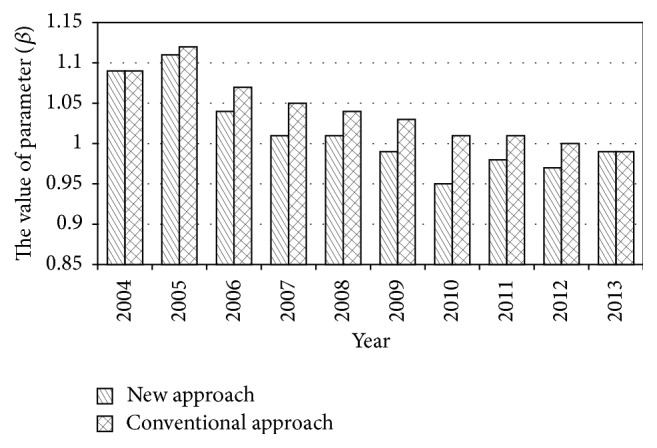
The value of parameter (*β*).

So, the value of parameter from 2004 to 2013 can be calculated by using this new approach. In order to compare the result of the new approach and the conventional approach, the updating results are listed in Table 2 from 2004 to 2013, and the comparison of the data in Table 2 is shown in Figures 2–4.

From the process of updating parameter, the AI-based approach for updating parameters realizes the parameter self-updating of bridge deterioration model when new data was collected, instead of depending on manual intervention, which is the biggest advantage of this approach. In addition, according to the case study results, it can be found that the results of new approach and conventional approach are approximately equal each year (i.e., the difference of the value of parameter *α* between new approach and conventional approach varies from 0 to 0.5 and the difference of the value of parameter *β* between new approach and conventional approach varies from 0 to 0.04). At the same time, the size of processed data is significantly reduced (i.e., the size of processed data decreases from 1725 to 182 in 2013) that would save much computational time and resource. Consequently, the AI-based approach for updating parameters in this paper is effective and practical for bridge deterioration model and gets rid of manual intervention.

## 4. Conclusions

Artificial intelligence is a computer science on the research and application of the law of the activities of human intelligence, and it has a broad application prospects in the practice of engineering. In bridge management, the information about the condition of bridge performance is increasing with time, so the parameter updating of bridge deterioration model is very critical for bridge management. To solve the problems of manual intervention and demanding for large computing resources of the conventional approach, an AI-based approach for updating parameters was presented. In this approach, the initial information is described by a prior distribution, and new information is viewed as a sample; then the posterior distribution is deduced, which can be used to update the deterministic model parameter. In addition, this approach is verified to be effective by practical data in a case study. The findings indicate that the new approach is a formal and mathematically consistent updating approach, which can be used to realize the parameter self-updating of bridge deterioration model without manual intervention and increase efficiency of updating parameters.

## Figures and Tables

**Figure 4:**
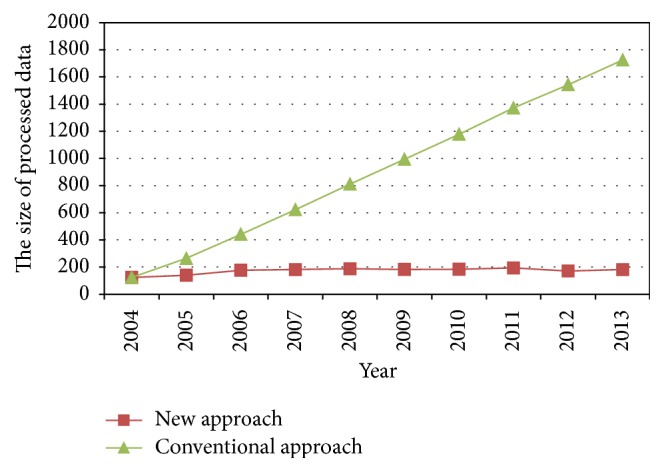
The size of processed data.

**Table 1:** Regressive results of new collected data.

Year	The size of processed data	The value of parameter (*α*)	The value of parameter (*β*)	The value of *δ* ^2^
2004	124	22.0	1.09	0.390
2005	140	21.6	1.14	0.316
2006	177	23.2	0.98	0.180
2007	182	23.3	0.98	0.190
2008	188	22.4	1.00	0.102
2009	183	22.7	0.99	0.169
2010	184	23.7	0.91	0.100
2011	194	22.9	1.00	0.142
2012	171	23.0	0.97	0.116
2013	182	23.0	0.95	0.184

**Table 2:** Results of new approach and conventional approach.

Year	The size of processed data		The value of parameter (*α*)		The value of parameter (*β*)	
	New	Conventional	New	Conventional	New	Conventional
2004	124	124	22.0	22.0	1.09	1.09
2005	140	264	21.8	21.7	1.11	1.12
2006	177	441	22.5	22.2	1.04	1.07
2007	182	623	22.9	22.4	1.01	1.05
2008	188	811	22.6	22.4	1.01	1.04
2009	183	994	22.7	22.5	0.99	1.03
2010	184	1178	23.2	22.7	0.95	1.01
2011	194	1372	23.1	22.7	0.98	1.01
2012	171	1543	23.0	22.8	0.97	1.00
2013	182	1725	23.0	22.8	0.99	0.99
